# The assessment of erectile dysfunction after radical prostatectomy using pudendal somatosensory evoked potential

**DOI:** 10.1371/journal.pone.0292847

**Published:** 2023-11-29

**Authors:** Se Yun Kwon, Jin-Mo Park

**Affiliations:** 1 Department of Urology, Dongguk University College of Medicine, Dongguk University Gyeongju Hospital, Gyeongju, Republic of Korea; 2 Department of Neurology, Dongguk University College of Medicine, Dongguk University Gyeongju Hospital, Gyeongju, Republic of Korea; General Sir John Kotelawala Defence University Faculty of Medicine, SRI LANKA

## Abstract

Erectile dysfunction in patients who underwent radical prostatectomy was evaluated with pudendal somatosensory evoked potentials (PSEP) to measure and predict erectile dysfunction objectively. Fifty-seven patients who completed requirements were included in the study. Patients were divided into 2 groups (potency/non-potency). Erectile function recovery was defined as question 2 and 3 on the IIEF-5 questionnaire at 12 months after surgery. The two-channel PSEP test was performed at the day before RP and 3–6 months after RP. Twenty patients were assigned to the potency group and 37 to the non-potency group. Mean age was less in the potency group. Other clinical variables were similar in two groups. The non-potency group had prolonged lumbar and cortical latencies in postoperative PSEP, and the mean differences of latencies between pre- and postoperative PSEP in lumbar and cortical regions were also greater in the non-potency group. Logistic regression analysis showed that age, lumbar post-operative latency, cortical post-operative latency, and difference of latency in lumbar region were associated with non-potency; odds ratios were 1.292 (*p* = 0.018), 0.425 (p = 0.047), 1.637 (p < 0.001), and 3.272 (p = 0.010), respectively. This study suggests that PSEP is an effective means of evaluating erectile dysfunction in prostate cancer patients after surgery.

## Introduction

Prostate cancer is the second most common cancer among men worldwide, accounting for roughly one-quarter of all male cancers [1–3]. According to national cancer registration statistics [4], prostate cancer has been more prevalent in South Korea recently as a result of an aging population and a westernized lifestyle [3]. In 2012, it was the fifth most common disease in men overall [4].

The frequency of radical prostatectomy (RP), the conventional treatment for prostate cancer, has continuously increased in line with the disease’s incidence [5]. Over the last two decades, surgical treatment of prostate cancer has significantly advanced, with excellent oncological and functional outcomes being achieved [6]. On the other hand, sexual dysfunction continues to be a prevalent issue, and owing to its influence on the quality of life in terms of health, attempts are being undertaken to lessen its impact [7]. Walsh et al., in the 1980s, before the adoption of the concept of nerve preservation during RP, reported postoperative erectile dysfunction in most patients [8]. Depending on the surgical techniques and patient selection methods used, nerve preservation has recently been found to retain erectile function in 29%–91% of patients [9, 10]. Furthermore, nerve preservation has been shown to reduce postoperative urinary incontinence [11].

The International Index of Erectile Function (IIEF) questionnaire or its abridged counterpart (IIEF-5) has been the main tool used to measure sexual dysfunction thus far. However, while these questionnaires help in identifying subjective symptoms, they fall short in obtaining an objective measure of sexual dysfunction. Many electrophysiological investigations have been performed to unbiasedly evaluate sexual dysfunction. For example, pudendal nerve somatosensory evoked potential (PSEP) studies are useful for diagnosing patients with possible pudendal afferent dysfunction [12]. Somatosensory evoked potentials are evoked potentials obtained by repeatedly stimulating peripheral nerves in the brain or spinal cord [13]. SEP has been routinely used over the years to evaluate the somatosensory pathway. PSEP assesses the integrity of afferent pathways leading from the pudendal nerve to the brain [12]. Many neurophysiological methods, including the pudendal nerve terminal motor delay, PSEP, perineal sympathetic skin response, perineal motor evoked potentials, needle electro-myography for the urethral sphincter, and bulbocavernosus reflex, can be used to study neurogenic urinary dysfunction [14–16]. Although there are many tests for evaluating urinary dysfunction, their exact standardization has not yet been established, and each test that evaluates distinct divisions of the nervous system has its strengths and limitations [14–16]. Even though PSEP is not the finest neurophysiological test, it has the benefit of being simple to use in hospitals with evoked potentials machines existing. PSEP covers most of the nervous system, and PSEP with more than two channels allows for the evaluation of detailed levels in the nervous system.

In this study, postoperative sexual dysfunction in individuals who had undergone radical prostatectomy was evaluated using two channels PSEP testing of the pudendal nerve.

## Materials and methods

### Study design and patient enrollment

This study mainly aimed to identify variables including PSEP results that predicted erectile dysfunction after 1 year of RP. Patients diagnosed with localized prostate cancer who underwent RP at our hospital between January 2017 and June 2019 were registered in this series after receiving approval from the regional institutional review board and ethics committee (110757-201808-HR-01-05). All participants provided written informed consent. RP with bilateral nerve-sparing was performed using the open technique [17]. The decision of applying nerve-sparing was mainly made based on the status of the tumor during surgery. Patients who fulfilled the qualifying requirements were signed; they completed the Korean version of IIEF-5 survey through self-administration [18] and underwent preoperative PSEP a day before surgery. PSEP was repeated at 3–6 months after surgery, and the IIEF-5 questionnaire was readministered at 12 months after surgery. Patients that achieved erectile function recovery were allocated to a ‘potency group’, and those with erectile dysfunction function post-RP were assigned to a ‘non-potency’ group. Low-dose (5 mg) phosphodiesterase type 5 inhibitors were administered to all RP patients who were not contraindicated for 3 months.

Patients who could not apply nerve sparing technique due to operational circumstance were excluded. Patients with existing erectile dysfunction before RP were also excluded. Patients with existing brain or spinal cord disorders which could affect the result of PSEP test were not included. In addition, patients who did not undergo the follow-up PSEP test were excluded.

### Definition of erectile function recovery and continence

According to questions 2 and 3 of the IIEF-5 questionnaire, erectile function recovery at 12 months following surgery was defined as the capacity to achieve penetration ≥50% of the time and maintain an erection strong enough for penetration ≥50% of the time. Patients who recovered their erectile function were assigned to a “potency group,” while those who developed erectile dysfunction after RP were assigned to a “non-potency” group. Continence was defined as a patient reporting no pad use or urine leakage at 12 months following surgery. Patients were asked the following question: “How many pads or adult diapers have you used daily to control leakage over the last 4 weeks?”

### Neurophysiological study of PSEP

We applied the two-channel PSEP test to evaluate the integrity of the nerve system from the penis to the brain cortex level twice (the day before RP and 3–6 months after RP). PSEP involved the placement of recording electrodes at the lumbar spine and cerebral cortex. A reference electrode was positioned at the top of the anterior iliac crest, and an active electrode was positioned at the L1 level in the lumbar region. According to the “10–20” system, a 5 mm cup active electrode was placed at the scalp midline, 2 cm behind the vertex at the Cz region, and a reference electrode was placed in the forehead midline at the Fz region, as previously described [19]. At the base of the penile shaft, stimulating bipolar ring electrodes with the anode distal and the cathode proximal were positioned. Patients were given square wave pulses with a duration of 0.1 ms, an intensity of 30 mA, and a frequency of 3 Hz. Over the gluteus maximus muscle, a ground electrode was positioned. Cortical and lumbar latencies were obtained by stimulating the pudendal nerve by averaging 250 responses.

### Clinical assessments

We obtained the following clinical data: age, body mass index (BMI), estimated blood losses (EBLs), pre- and postoperative IIEF-5 scores, pre- and postoperative PSEP latencies, prostate volume, operation time, and complications. In addition, we analyzed preoperative prostate-specific antigen (PSA) levels, postoperative Gleason scores, pathologic stages, and biochemical recurrences (BCR). A serum PSA level of > 0.2 ng/mL at two successive assessments performed 1 year after RP was used to define BCR.

### Statistical analysis

This prospective study was conducted based on our previous retrospective study using data from 2014–2015 [20]. And the sample size to verify the difference between the mean of PSEP between case and control was calculated using open source software [21]. We set group ratio = 3 (33/11) and the mean value of each group is 16.9 ± 1.7 / 19.4 ± 2.0 from previous study (with 95% confidence interval and 0.8 power), it seems that at least 8 people in group 1 (potency) and at least 18 subjects should be included in group 2 (non-potency) to verify that the difference of PSEP between two groups is significant (with alpha = 0.05 and beta = 0.8). To account for potential attrition, we increased the sample sizes by 10%. This results in a revised minimum of 9 subjects for the potency group and 20 subjects for the non-potency group.

Normality of variables was determined using the Kolmogorov–Smirnov test. Continuous and categorical variables were compared using independent Student’s t-test or non-parametric Mann Whitney U-test, and Chi-square test. Pearson correlation coefficients were used to assess the relationships between clinical parameters and neurophysiological findings. Utilizing logistic regression (forward selection (likelihood ratio)) analysis, the relative risk of characteristics that significantly differed between the potency and non-potency groups was evaluated. The statistical analysis was performed using PASW^®^ Statistics ver. 18.0 (SPSS Inc., Chicago, Ill., USA), and p values of < 0.05 were used to define statistical significance.

## Results

### Patient characteristics

**Fig 1:**
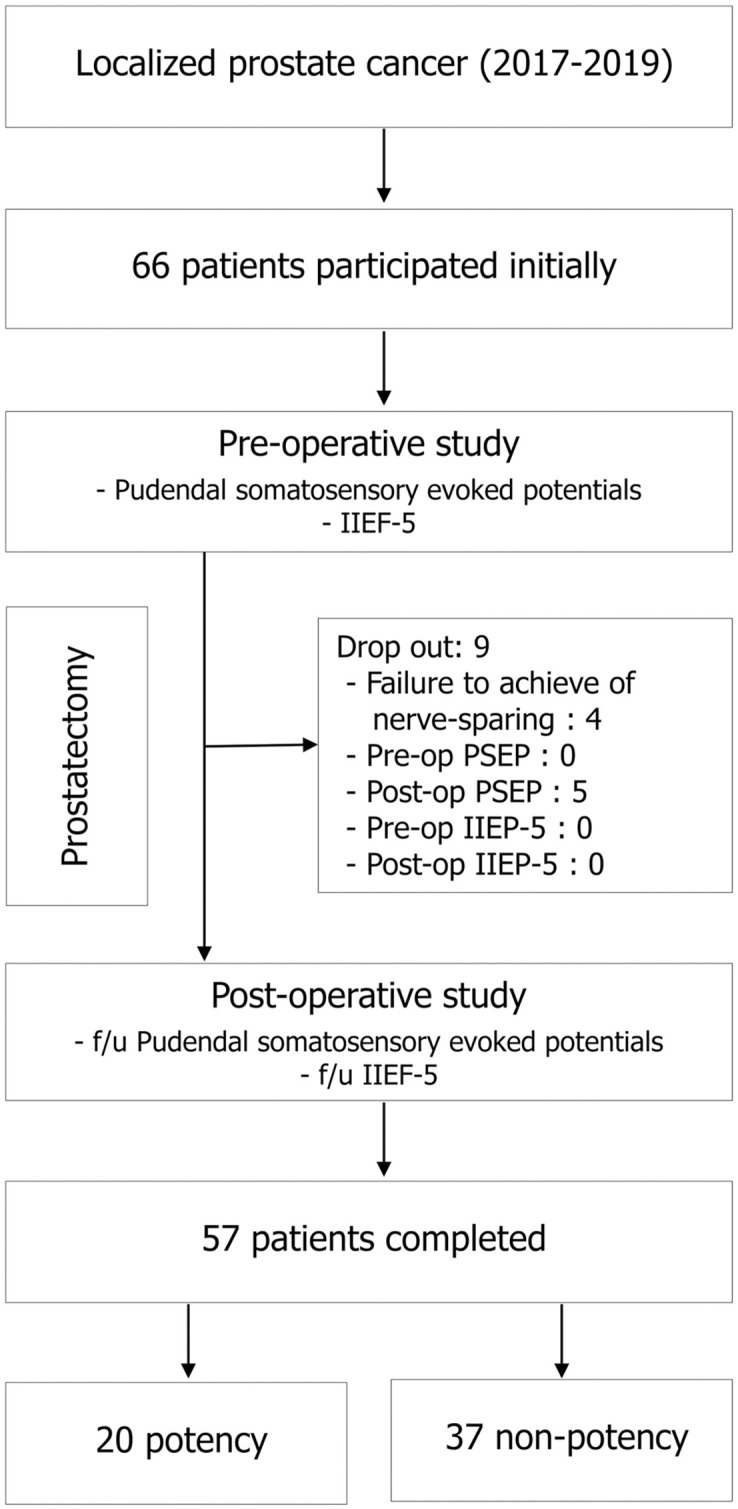
A flow diagram of the prostate cancer patients with nerve-sparing surgery.

A total of 66 consecutive patients who agreed informed consents was enrolled. 9 patients were excluded owing to failure to achieve nerve-sparing and incomplete of PSEP (Fig 1). The clinical data of the 57 patients who completed the qualifying requirements was analyzed. The average age of the 57 patients was 68.8 years (67–70 range), with a mean BMI of 24.5 ± 2.8 kg/m² and prostate volume of 40.7 ± 18.7 ml. Pre-operative PSA averaged 10.24 ± 8.8 ng/ml, with six patients exceeding 20 ng/ml. Pathologic organ-confined disease was found in 37 patients (64.9%). Operation time and estimated blood loss averaged 168 ± 38 min and 284.0 ± 134.1 ml, respectively. There was no association between clinical variables, except for a relationship between BMI and operation time (r = 0.396, p = 0.002). The mean duration between the preoperative and postoperative PSEP was 5.1 ± 1.5 months.

### Comparison of potency and non-potency groups

At 12 months after RP, 20 patients were assigned to the potency group, while 37 were assigned to the non-potency group (Table 1). The average age of the potency group was lower than that of the non-potency group (66.8 ± 5.5 years vs. 69.8 ± 5.2 years, p = 0.039). Other clinical factors such as the mean prostate volume, BMI, mean operation time, EBLs, transfusion dose (ml), and complication rates were comparable across the two groups. Only one patient in the non-potency group experienced a perioperative complication and postoperative bleeding, which was successfully managed conservatively. Preoperative PSA levels did not significantly differ between groups. In the potency group, 13 patients had pathologic stage T2 and 7 had stage T3, while in the non-potency group, 20 had stage T2 and 17 had stage T3. The two groups had similar mean Gleason scores and lymphatic/vascular invasion rates as well as good surgical margins. BCR was also similar between the two groups. A total of 53 patients had attained urinary continence at 12 months after RP, compared to 4 patients of urinary incontinence (1 in the potency group and 3 in the non-potency group), with no discernible intergroup differences (p = 0.643). The number of patients who administered phosphodiesterase type 5 inhibitors for 3 months were not significantly higher in the potency group (90.0% vs. 64.8%, p = 0.059), and the total IIEF-5 score before RP was similar in the two groups (p = 0.074). Table 1 provides a summary of the clinical characteristics of the two groups.

**Table 1:** Characteristic features of the potency and non-potency groups.

		Potency (n = 20)	Non-potency (n = 37)	p value
Age (years), mean (sd)		66.8 ± 5.5	69.8 ± 5.2	**0.039**
Body mass index (kg/m^2^), mean (sd)		24.6 ± 2.6	24.3 ± 3.0	0.675
Prostate volume (ml), mean (sd)		40.8 ± 17.1	40.7 ± 19.8	0.860
Preoperative PSA (ng/ml), no. (%)				0.079
<10		16 (80.0)	21 (56.8)	
>10		4 (20.0)	16 (43.2)	
Mean operation time (min), mean (sd)		171.3 ± 42.2	167.6 ± 36.1	0.731
Estimated blood loss (ml), mean (sd)		280.8 ± 158.2	265.7 ± 121.5	0.633
Number of complications, no. (%)		0 (0.0)	1 (2.7)	0.458
Catheterization (day), mean (sd)		14.7 ± 7.6	16.7 ± 7.7	0.204
Pathologic stage, no. (%)				0.424
T2		13 (65.0)	20 (54.1)	
T3		7 (35.0)	17 (45.9)	
Pathologic Gleason score, no. (%)				0.227
6~7		8 (40.0)	21 (56.8)	
8~10		12 (60.0)	16 (43.2)	
Positive surgical margin, no. (%)		5 (25.0)	13 (35.1)	0.432
Biochemical recurrence, no. (%)		2 (20.0)	8 (21.6)	0.271
Patients administered phosphodiesterase type 5 inhibitors for 3 months, no. (%)		18 (90.0)	24 (64.9)	0.059
Duration of pre- and post-study (month), mean (sd)		4.6 ± 1.7	5.3 ± 1.3	0.163
International Index of Erectile Function-5 score, mean (sd)	Pre-op	14.0 ± 7.0	10.4 ± 6.9	0.074
	Post-op	11.8 ± 6.9	3.2 ± 3.4	**<0.001**
Incontinence after 1 year, no. (%)		1 (5.0)	3 (8.1)	0.643

Continuous variables were analyzed using an independent Student’s t-test, while categorical variables were evaluated using a Chi-square test.

### Results of pudendal somatosensory evoked potentials

In all study participants (57 patients), before RP, the mean latencies in the lumbar and cortical regions were 12.8 ± 1.2 ms and 47.6 ± 3.0 ms, respectively, whereas after RP, these values were 17.0 ± 2.6 ms and 51.7 ± 3.4 ms, respectively. And the mean difference of latency between pre- and post-RP PSEP in each lumbar and cortical region was 4.3 ± 2.8 ms and 4.0 ± 3.2 ms, respectively. PSEP latencies did not show a statistically significant correlation with age, BMI, PSA level, operation time, or pathologic Gleason score. However, a notable exception was the relationship between estimated blood losses and post-RP lumbar latency (Table 2).

**Table 2:** Correlations between PSEP results and clinical variables.

		Pudendal somatosensory evoked potentials					
		Pre-RP latency		post-RP latency		Difference (Δ)	
		Lumbar	Cortical	Lumbar	Cortical	Lumbar	Cortical
Age	r	0.168	0.067	-0.009	0.033	-0.168	-0.012
	p	0.212	0.619	0.947	0.805	0.210	0.929
BMI	r	-0.169	0.244	-0.043	0.141	0.115	0.001
	p	0.208	0.067	0.749	0.295	0.394	0.996
Preoperative PSA	r	0.191	0.086	0.041	0.025	-0.138	-0.010
	p	0.155	0.523	0.764	0.852	0.305	0.942
Operation time	r	-0.023	0.004	-0.197	-0.219	-0.188	-0.214
	p	0.868	0.974	.0.141	0.102	0.162	0.110
Estimated blood losses	r	0.201	0.005	0.338	0.230	0.167	0.191
	p	0.134	0.973	**0.010**	0.085	0.216	0.156
Pathologic Gleason score	r	-0.024	0.123	0.039	0.207	0.063	0.141
	p	0.861	0.362	0.774	0.122	0.644	0.295

r: Pearson correlation coefficient.

The mean duration between the preoperative and postoperative PSEP was not differ significantly in potency and non-potency groups (4.6 ± 1.7 months vs. 5.3 ± 1.3 months, p = 0.163). Comparing the PSEP results in the potency and non-potency groups (Table 3), patients in the non-potency group had significantly prolonged lumbar and cortical latencies of the postoperative PSEP than those in the potency group (lumbar region: 17.5 ± 2.4 ms vs. 15.1 ± 2.6 ms, p = 0.043 and cortical region: 53.0 ± 2.7 ms vs. 49.2 ± 3.2 ms, p < 0.001), and the mean difference of latency (Δ latencies) between pre- and post-RP PSEP in each lumbar and cortical region was also significantly greater in the non-potency group than those in the potency group (lumbar region: 5.1 ± 2.8 vs. 2.9 ± 2.4 ms, p = 0.006 and cortical region: 5.1 ± 2.8 vs. 2.3 ± 3.2 ms, p < 0.001).

**Table 3:** Results of pudendal somatosensory evoked potentials.

	Overall (n = 57) mean (sd)	Potency (n = 20) mean (sd)	Non-potency (n = 37) mean (sd)	p value
**Preoperation**				
Lumbar latency (ms)	12.8 ± 1.2	12.3 ± 1.2	12.5 ± 1.1	0.260
Cortical latency (ms)	47.6 ± 3.0	46.9 ± 3.1	47.9 ± 3.1	0.237
**Postoperation**				
Lumbar latency (ms)	17.0 ± 2.6	15.1 ± 2.6	17.5 ± 2.4	**0.043**
Cortical latency (ms)	51.7 ± 3.4	49.2 ± 3.2	53.0 ± 2.7	**<0.001**
**Difference (pre and post)**				
Δ-Lumbar latency (ms)	4.3 ± 2.8	2.9 ± 2.4	5.1 ± 2.8	**0.006**
Δ-Cortical latency (ms)	4.0 ± 3.2	2.3 ± 3.2	5.1 ± 2.8	**<0.001**

These variables (age, lumbar and cortical postoperative latency and the Δ latency in lumbar and cortical region) that significantly differed (p < 0.05) in the two groups according to univariate analysis were used to determine risk factors for postoperative non-potency using multivariable logistic regression analysis. Age, lumbar postoperative latency, cortical postoperative latency, and the Δ latency in lumbar region were revealed as risk factors of non-potency; the odds ratios were 1.292 (1.045–1.596, p = 0.018), 0.425 (0.182–0.989, p *=* 0.047), 1.637 (1.241–2.160, p < 0.001), and 3.272 (1.325–8.081, p = 0.010), respectively (Table 4). To assess the absence of multicollinearity among the predictors, a separate linear regression analysis was conducted using the same variables as in the logistic regression model. The variance inflation factors for each factor were found to be below the common threshold of 10 (1.071 for age, 1.554 for lumbar postoperative latency, 4.618 for cortical postoperative latency, 1.956 for Δ latency in the lumbar region, and 4.305 for Δ latency in the cortical region). Additionally, the pseudo R-squared values of Nagelkerke was 0.643.

**Table 4:** Results of multivariable logistic regression analysis.

	B	SE	Wald χ2	p	OR	95% C.I.
Age	0.256	0.108	5.618	0.018	1.292	1.045–1.596
Lumbar postoperative latency	-0.857	0.431	3.943	0.047	0.425	0.182–0.989
Cortical postoperative latency	0.493	0.141	12.137	<0.001	1.637	1.241–2.160
Δ-Lumbar latency	1.185	0.461	6.601	0.010	3.272	1.325–8.081

B: The regression coefficient, SE: Standard error of the regression coefficient, Wald χ2: Wald Chi-square statistic, p: The p-value associated with the Wald Chi-square statistic, OR: odd ratio or exponentiated coefficient (Exp(B)), 95% C.I.: The 95% confidence interval for the odds ratio. Pseudo R-Squared (Nagelkerke): 0.643

## Discussion

In this study, PSEP revealed significant differences of results between RP patients with and without erectile dysfunction. The postoperative latencies of non-potency group in the lumbar and cortical regions were both noticeably prolonged. In addition, the logistic regression analysis revealed risk factors of erectile dysfunction after RP. Older age, prolonged cortical latency of postoperative PSEP and the greater difference of latency between pre- and postoperative PSEP in lumbar region were significantly associated with erectile dysfunction. Our logistic regression analysis has elucidated the salient risk factors for erectile dysfunction following RP, such as advanced age, protracted postoperative cortical latency of PSEP, and a pronounced disparity in latency between pre- and postoperative PSEP in the lumbar region. Notably, a greater differential in lumbar latency had the highest OR. Interestingly, an inverse relationship was observed between lumbar postoperative latency and erectile dysfunction in the multivariable analysis, whereas a direct relationship was evident for cortical postoperative latency. This apparent contradiction, given the elevated lumbar and cortical postoperative latencies in the non-potency group, warrants consideration of covariate influences within the multivariable model. This could be a manifestation of varying physiological factors or disparities in nerve recovery and conduction between lumbar and cortical regions. Moreover, lumbar postoperative latency might not precisely reflect the extent of pudendal nerve damage and may interact with other model variables, like age and pre- and postoperative latency differentials, in a multifaceted manner. A more thorough investigation is warranted in future research to decode these intricate relationships and their roles in forecasting post-RP erectile function. Meanwhile, it is imperative to recognize the value of pre- and postoperative PSEP as reliable predictive tools for post-RP outcomes and to incorporate them into standard assessment protocols. Our study uncovers a noteworthy correlation between EBLs and lumbar latency following RP, while showing no correlation with latency difference between pre- and post-RP PSEP in lumbar region. This observation prompts several key questions. Firstly, the elevated EBL may suggest a more complex surgery, possibly affecting post-RP lumbar latency through mechanisms like altered neural pathways or postoperative inflammation. Conversely, the lack of correlation between EBL and latency differences in lumbar region raises the possibility that preoperative nerve conditions could mask any effects of EBL on latency. Secondly, it raises the critical issue of whether this correlation is genuinely causal or influenced by other unmeasured variables. These findings highlight the need for further research to clarify the relationship between EBL and lumbar latency, its clinical implications, and its potential impact on other postoperative outcomes.

We performed the second PSEP study at 5.13 ± 1.45 months and evaluated patients’ erectile function after 12 months from surgery. Thus, PSEP can help determine the prognosis of a patient’s erectile function after RP. It is not yet known when these differences could be determined and measurable. Further study needs to clarify that when the difference can be identified through the PSEP after surgery in predicting the patient’s prognosis earlier.

Patients in the potency group tended to be younger, which is consistent with previous studies that found younger patients had excellent erectile function preservation after RP [22]. Therefore, younger patients and those with less pudendal nerve damage, as measured by PSEP, appear to have better erectile function. At this point, the question arises as to how the degree of damage to the pudendal nerve could reflect erectile function. It is known that RP-related impotence is brought on by injury to the neurovascular bundle, specifically the cavernous nerve, which is situated in the posterior region of the prostate during prostate removal [23]. Furthermore, penile hemodynamic abnormalities caused by vasculogenic components during surgery result in significant tissue damage, such as increased corporeal fibrosis, which influences the occurrence of impotence [24]. In the process of removing the prostate and surrounding tissue, it is assumed that the pudendal nerve is damaged in proportion to the damage to the neurovascular bundle.

RP is a relatively sophisticated procedure that is performed in a confined space, and recently, robot-assisted laparoscopy has produced good results in terms of postoperative pain relief and bleeding loss [25], other studies have also reported its lower incidences of erection dysfunction and incontinence [26–28]. In this study, we used an open procedure to perform RP, and the overall rates of non-potency and incontinence at 12 months were 62.3% and 7.0%, respectively, which are comparable to those re-ported for robotic surgery cases.

Recently, as interest in preserving erectile function after surgery has increased as a process of improving quality of life, various electrophysiologic techniques such as enhancing the preservation of neurovascular bundles through intraoperative monitoring are currently being used [29]. Nonetheless, PSEP has an advantage of easily applicable device, and it is hoped that further confirmation of its efficacy will be made in the future.

## Conclusions

This study showed significant findings of neurophysiologic study between RP patients with or without erectile dysfunction. PSEP can be an objective tool for assessing erectile dysfunction in prostate cancer patients after surgery. Further studies are warranted to evaluate whether it can predict postoperative erectile function.
